# Pulmonary fibrosis and its related factors in discharged patients with new corona virus pneumonia: a cohort study

**DOI:** 10.1186/s12931-021-01798-6

**Published:** 2021-07-09

**Authors:** Xiaohe Li, Chenguang Shen, Lifei Wang, Sumit Majumder, Die Zhang, M. Jamal Deen, Yanjie Li, Ling Qing, Ying Zhang, Chuming Chen, Rongrong Zou, Jianfeng Lan, Ling Huang, Cheng Peng, Lijiao Zeng, Yanhua Liang, Mengli Cao, Yang Yang, Minghui Yang, Guoyu Tan, Shenghong Tang, Lei Liu, Jing Yuan, Yingxia Liu

**Affiliations:** 1 Shenzhen Key Laboratory of Pathogen and Immunity, National Clinical Research Center for Infectious Disease, State Key Discipline of Infectious Disease, Shenzhen Third People’s Hospital, Second Hospital Affiliated to Southern University of Science and Technology, No. 29, Bulan Road, Longgang district, Shenzhen, 518112 China; 2 Department of Radiology, Shenzhen Third People’s Hospital, Second Hospital Affiliated to Southern University of Science and Technology, Shenzhen, China; 3 School of Public Health, Southern Medical University, Guangzhou, 510515 China; 4 EEE Department, Southern University of Science & Technology, China and ECE Department, McMaster Univ., Hamilton, Canada

**Keywords:** Persistent consequences, Pulmonary fibrosis, Risk factor, COVID-19 recovered patient, Lung function

## Abstract

**Background:**

Thousands of Coronavirus Disease 2019 (COVID-19) patients have been discharged from hospitals Persistent follow-up studies are required to evaluate the prevalence of post-COVID-19 fibrosis.

**Methods:**

This study involves 462 laboratory-confirmed patients with COVID-19 who were admitted to Shenzhen Third People’s Hospital from January 11, 2020 to April 26, 2020. A total of 457 patients underwent thin-section chest CT scans during the hospitalization or after discharge to identify the pulmonary lesion. A total of 287 patients were followed up from 90 to 150 days after the onset of the disease, and lung function tests were conducted about three months after the onset. The risk factors affecting the persistence of pulmonary fibrosis were identified through regression analysis and the prediction model of the persistence of pulmonary fibrosis was established.

**Results:**

Parenchymal bands, irregular interfaces, reticulation and traction bronchiectasis were the most common CT features in all COVID-19 patients. During the 0–30, 31–60, 61–90, 91–120 and > 120 days after onset, 86.87%, 74.40%, 79.56%, 68.12% and 62.03% patients developed with pulmonary fibrosis and 4.53%, 19.61%, 18.02%, 38.30% and 48.98% patients reversed pulmonary fibrosis, respectively. It was observed that Age, BMI, Fever, and Highest PCT were predictive factors for sustaining fibrosis even after 90 days from onset. A predictive model of the persistence with pulmonary fibrosis was developed based-on the Logistic Regression method with an accuracy, PPV, NPV, Sensitivity and Specificity of the model of 76%, 71%, 79%, 67%, and 82%, respectively. More than half of the COVID-19 patients revealed abnormal conditions in lung function after 90 days from onset, and the ratio of abnormal lung function did not differ on a statistically significant level between the fibrotic and non-fibrotic groups.

**Conclusions:**

Persistent pulmonary fibrosis was more likely to develop in patients with older age, higher BMI, severe/critical condition, fever, a longer viral clearance time, pre-existing disease and delayed hospitalization. Fibrosis developed in COVID-19 patients could be reversed in about a third of the patients after 120 days from onset. The pulmonary function of less than half of COVID-19 patients could turn to normal condition after three months from onset. An effective prediction model with an average area under the curve (AUC) of 0.84 was established to predict the persistence of pulmonary fibrosis in COVID-19 patients for early diagnosis.

## Background

The Coronavirus Disease 2019 (COVID-19) caused by the severe acute respiratory syndrome coronavirus 2 (SARS-CoV-2) has become a pandemic [1]. As of June 12, 2021, there have been 174,918,667 confirmed cases of COVID-19, including 3,782,490 deaths globally, posing a serious threat to public health worldwide [2]. Infection of SARS-CoV-2 may cause atypical pneumonia with clinical presentation, mainly causing respiratory system infections in humans ranging from minor common cold to severe diseases [1, 3]. COVID-19 causes pulmonary syndromes similar to other strains of the coronavirus family, namely severe acute respiratory syndrome (SARS) coronavirus and Middle East respiratory syndrome (MERS) coronavirus. Previous data from the study of MERS and SARS suggested that there could be substantial fibrotic consequences following SARS-CoV-2 infection [4, 5].

Chest computed tomography (CT) plays an important role in the diagnosis and treatment of patients with COVID-19 that helps in the diagnosis by depicting lung abnormalities and in the evaluation of the progress of disease and response to treatment [6–8]. Several studies have described the imaging and clinical features of serial thin-section CTs in COVID-19 patients during hospitalization or discharged after treatment [9–14]. At present, it is not known if COVID-19 patients who survived pneumonia would be at risk of chronic sequelae. Persistent follow-up studies are required to evaluate the prevalence of post-COVID-19 fibrosis. In this study, by observing the persistent dynamic changes and the predictors of pulmonary fibrosis in discharged patients with COVID-19, we tried to identify whether the development of persistent pulmonary fibrosis occurs in the survivor population and find early warning indicators that are linked to pulmonary fibrosis, to develop an early intervention tool to reduce the occurrence of pulmonary fibrosis.

## Methods

### Study design and patients

This study involves 462 patients with COVID-19 who had been admitted to Shenzhen Third People’s Hospital from January 11, 2020 to April 26, 2020. COVID-19 patients were diagnosed using quantitative reverse-transcription polymerase chain reaction (qRT-PCR) based on the World Health Organization's interim guidance [15]. Among them, a total of 457 patients underwent thin-section chest CT scans during the hospitalization or after discharge. These patients were discharged from the hospital between January 23, 2020 and May 21, 2020, and the discharge criteria were in line with the Chinese guideline for COVID-19 pneumonia. Only one death was included in this study, this patient died 44 days after onset. COVID-19 patients who did not undergo thin-section chest CT scans were excluded from this study.

After discharge, the patients were followed up every four weeks or so. A Thin-section chest CT scan was performed for each patient at each visit. However, only a part of the patients underwent laboratory tests at the follow-up visit because of the insufficient medical resources during the pandemic. The final date of follow-up was June 20, 2020. The average observation time of these 457 patients was 80.57 days after onset. The median observation time was 76 days, with the shortest and the longest being 12 and 151 days, respectively. A total of 289 patients were followed up from 90 to 150 days after the onset of the disease. During the period of hospitalization, 457 patients underwent routine laboratory tests every 3 to 7 days, and CT scans every 3–5 days. Besides, lung function tests were conducted in about 3 months after the onset. Clinical data during hospitalization, and imaging and pulmonary function data after discharge of these patients were obtained by reviewing their pulmonary fibrosis status at different times from the hospital’s computerized medical record system. The effect of pulmonary fibrosis on pulmonary function was analyzed simultaneously. The risk factors affecting the persistence of pulmonary fibrosis were identified through regression analysis and a model to predict the persistence of pulmonary fibrosis was established.

### CT imaging

Several non-contrast thin-section chest CT scans were performed for each patient using two independent medical CT machines. Equipment and scanning parameters are as follows: (1) Toshiba TSX-101A64 row spiral CT machine (tube voltage 120 kV, automatic tube current, reconstructed layer thickness 1 mm). (2) Shanghai uCT760 64-row spiral CT machine (tube voltage 120 kV, automatic tube 40 mA, reconstructed layer thickness 0.625 mm). All image data were observed in the pulmonary window, with window width and window level of 1600 HU and − 550 HU, respectively.

### Judgment of pulmonary fibrosis and the resolution of pulmonary fibrosis

All thin-section CT images were independently analyzed by three experienced radiologists to determine the presence of pulmonary fibrosis, and any disagreement was resolved by discussion and consensus. Pulmonary fibrosis on chest CT imaging was defined as a combination of findings including parenchymal bands, irregular interfaces, reticulation and traction bronchiectasis [5, 16, 17]. The resolution of pulmonary fibrosis was evaluated by examining the patient's lung CT for the disappearance of the signs of original fibrosis.

### Disease severity classification

Disease severity classification and Murray Score calculation were evaluated as previously reported [18]. The severity of COVID-19 was graded according to the China National Health Commission Guidelines for Diagnosis and Treatment of SARS-CoV-2 infection. Laboratory confirmed patients with fever, respiratory manifestations and radiological findings indicative of pneumonia were considered as the moderate cases. Laboratory confirmed patients with any of the following conditions were considered to have severe COVID-19: (i) respiratory distress (respiration rate ≥ 30/min; (ii) resting oxygen saturation ≤ 93%, and (iii) arterial oxygen partial pressure (PaO_2_) / fraction of inspired oxygen (FiO_2_) ≤ 300 mmHg (1 mmHg = 0.133 kPa). Laboratory confirmed patients with any of the following conditions, such as (i) respiratory failure requiring mechanical ventilation, (ii) shock, and (iii) failure of other organs requiring intensive care unit (ICU), were considered to be in critical condition.

### Statistical analysis

Continuous variables were presented as mean ± standard deviation and categorical variables were presented as n (%). Event frequencies were compared with the Chi-square test, whereas the One-way Analysis of Variance (ANOVA) method was used to compare the continuous parameters between two groups. ANOVA provides a measure of statistical significance (p-value) in the difference between the distributions of a parameter in two groups. A p-value < 0.05 was considered statistically significant. Only a subset of statistically significant variables (p < 0.05) was considered to develop a simple logistic regression model–based clinical tool for early diagnosis. Variables with p > 0.05 were not considered for parameter selection and model development. The performance of the model was validated using a five-fold cross validation and assessed with the Receiver Operating Characteristic (ROC) curve, accuracy, Positive Predictive Value (PPV), Negative Predictive Value (NPV), Sensitivity and Specificity. The analysis was performed using a custom-written code in the MATLAB (version R2017b) environment. MATAB is a powerful and commonly used software for mathematical analyses, modeling, classification and prediction.

## Results

### Comparison of clinical characteristics between patients with pulmonary fibrosis and with no fibrosis/ resolution of pulmonary fibrosis after 90 days from onset

Our study included all 457 confirmed COVID-19 cases admitted to the Shenzhen Third People’s Hospital and followed up till June 20, 2020. To observe the persistent pulmonary consequences of COVID-19 patients, 289 confirmed COVID-19 patients who were followed up more than 90 days after onset, were further divided into two groups (group A and group B) according to the progression of pulmonary fibrosis. Group A (GA) had 116 (40.14%) patients who either had no lung fibrosis, or their lung fibrosis disappeared within 90 days after onset. On the other hand, 173 (59.86%) patients who still had lung fibrosis after 90 days from onset, were categorized as Group B (GB) (Table 1).

**Table. 1:** Epidemiological and baseline clinical features of 289 patients who were followed up from 90 to 150 days after the onset

	Group A^a^ (N = 116)	Group B^b^ (N = 173)	P value
Physical characteristics			
Gender (male ratio, %)	44%	52%	0.2212
Age (year)	33.06 ± 17.50	50.68 ± 13.25	< 0.0001
BMI (kg/m^2^)	22.10 ± 3.43	24.06 ± 3.21	< 0.0001
Pre-existing conditions (with pre-existing disease ratio)			
Pre-existing disease (%)	19.0%	38.2%	0.0008
DM (%)	0%	7.5%	0.0063
Hypertension (%)	3.5%	19.1%	0.0002
Coronary heart disease (%)	3.5%	6.9%	0.3131
Respiratory disease (%)	3.5%	5.2%	0.6776
Others			
Case type: (severe/critical ratio, %)	4.3%	29.5%	< 0.0001
Time from onset to virus RNA negative (day)	14.72 ± 8.22	20.99 ± 9.34	< 0.0001
Time from onset to admission (day)	3.01 ± 3.03	5.22 ± 4.29	< 0.0001
Length of hospitalization (day)	19.17 ± 6.85	24.35 ± 9.70	< 0.0001
Follow-up period from onset (day)	67.78 ± 36.09	117.77 ± 14.24	< 0.0001
Symptoms			
Fever (%)	51.7%	72.8%	0.0004
Fatigue (%)	9.5%	10.4%	0.9554
Dry cough (%)	18.1%	25.4%	0.1871
Cough (%)	13.8%	18.5%	0.3724
Expectoration (%)	9.5%	6.9%	0.5739
Diarrhea (%)	0.9%	5.8%	0.0675
Treatment			
Short term methylprednisolone therapy (%)	5.17%	32.3%	< 0.0001
Acetylcysteine (100%)	41.4%	71.7%	< 0.0001
Gamma globulin (100%)	4.3%	31.8%	< 0.0001
Invasive ventilator (100%)	0%	5.2%	0.0315
Non-invasive ventilator (100%)	0.9%	14.5%	0.0002
Laboratory findings			
Oxygenation index (PaO_2_/FiO_2_)	463.15 ± 123.05	411.60 ± 130.24	0.0014
Lactic acid (mmol/L)	1.29 ± 0.45	1.34 ± 0.44	0.2963
WBC (10^9^/L)	5.179 ± 2.04	4.82 ± 1.71	0.1174
N (10^9^/L)	2.85 ± 1.47	3.00 ± 1.43	0.3689
L (10^9^/L)	1.7659 ± 0.88	1.32 ± 0.59	< 0.0001
HB (g/L)	137.11 ± 15.43	138.28 ± 15.08	0.5240
PLT (10^9^/L)	210.35 ± 65.32	188.19 ± 56.95	0.0025
ALB (g/L)	43.91 ± 3.18	42.64 ± 3.55	0.0021
ALT (U/L)	24.54 ± 26.74	26.32 ± 17.20	0.4918
AST (U/L)	28.34 ± 20.18	31.01 ± 13.97	0.1855
LDH (U/L)	267.37 ± 144.00	308.43 ± 191.53	0.0594
CK (U/L)	115.30 ± 194.97	103.05 ± 112.20	0.5729
BUN (mmol/L)	4.16 ± 1.41	4.09 ± 1.62	0.6789
Cr (μmol/L)	60.92 ± 17.81	68.75 ± 20.66	0.0010
ESR (mm/h)	23.01 ± 20.32	35.95 ± 23.00	< 0.0001
D-Dimer (μg/mL)	0.42 ± 0.37	0.52 ± 0.68	0.1732
CRP (mg/dL)	12.25 ± 25.58	23.96 ± 30.38	0.0008
IL-6 (ng/L)	11.27 ± 19.57	20.68 ± 30.70	0.0112
PCT (ng/mL)	0.12 ± 0.10	0.09 ± 0.08	0.0053
CD4 cells (count/μL)	771.89 ± 419.79	535.68 ± 276.76	< 0.0001
Highest CRP (mg/dL)	19.23 ± 30.71	47.81 ± 54.94	< 0.0001
Highest IL-6 (ng/L)	8.91 ± 11.92	45.62 ± 142.25	0.0099
Highest PCT (ng/mL)	0.29 ± 0.09	0.32 ± 0.08	0.0003
Lowest CD4 cell (count/μL)	700.81 ± 383.69	471.46 ± 274.76	< 0.0001
Highest ESR (mm/h)	32.00 ± 29.07	50.29 ± 30.70	< 0.0001
Highest D-Dimer (μg/mL)	0.63 ± 0.84	1.40 ± 3.11	0.0095
Highest lactic acid (mmol/L)	2.19 ± 0.93	2.62 ± 0.85	0.0001
Lowest oxygenation index (PaO_2_/FiO_2_)	379.97 ± 118.46	270.93 ± 124.19	< 0.0001

*BMI* body mass index, *DM* diabetes mellitus, *PaO*_*2*_ partial pressure of oxygen, *FIO*_*2*_ fraction of inspired oxygen, *WBC* white blood cells, *N* neutrophils, *L* lymphocyte, *HB* hemoglobin, *PLT* platelet, *ALB* albumin, *ALT* alanine transaminase, *AST* aspartate aminotransferase, *LDH* lactate dehydrogenase, *CK* creatine kinase, *BUN* urea nitrogen, *Cr* creatinine, *ESR* erythrocyte sedimentation rate, *CRP* C-reactive protein, *IL-6* interleukin-6, *PCT* procalcitonin

^a^Patients who either had no lung fibrosis, or their lung fibrosis disappeared within 90 days after onset

^b^Patients who still had lung fibrosis after 90 days from onset

Among the physical characteristics studied, Age and BMI were found to be two statistically significant (p < 0.05) risk factors between the two groups. Older patients (mean age 50.68 years vs 33.06 years) and patients with higher BMI (mean BMI 24.1 kg/m^2^ vs 22.1 kg/m^2^) still showed signs of lung fibrosis even after 90 days from onset. Most symptom profiles were comparable between GA and GB. However, a higher proportion of patients in GB had fever compared to GA (~ 73% in GB vs ~ 52% in GA) which is statistically significant at p < 0.0001. Besides, severe/critical COVID-19 patients, as well as patients with pre-existing health conditions, were observed to be more vulnerable to sustaining lung fibrosis even after 90 days (Table 1).

It can also be seen that patients in the GB took significantly longer time (21 days vs 14.7 days, p < 0.0001) from onset to get virus RNA negative, required a longer follow-up period from onset (117.8 days vs 67.8 days, p < 0.0001), stayed in the hospital for a longer period (24.4 days vs 19.2 days, p < 0.0001), and more patients in GB receive antiviral treatment. These observations in the temporal patterns can be attributed to the significantly higher number (29.5% vs 4.3%, p < 0.0001) of severe/critical COVID-19 cases in GB, since severe/critical COVID-19 patients generally require treatment and follow-up for a longer period. It is interesting to see that an average delay of ~ 2 days before hospital admission following the onset of the symptoms had a significant (p < 0.0001) detrimental effect on the patients with pneumonia, thus rendering them more vulnerable to developing and sustaining lung fibrosis for a longer period (Table 1).

Levels of lactic acid, white blood cells (WBC), neutrophils (N), lymphocyte (HB), alanine transaminase (ALT), aspartate aminotransferase (AST), lactate dehydrogenase (LDH), creatine kinase (CK), urea nitrogen (BUN), and D-Dimer did not differ on a statistically significant level (*p* > 0.05) between the two groups (Table 1). However, there were statistically significant differences in some of the laboratory findings between the two groups. These differences (GB vs GA) include Lowest oxygenation index (PaO_2_/FiO_2_), Lowest CD4 cell, L (lymphocyte), CD4 cells, Highest CRP (C-reactive protein), Highest ESR (erythrocyte sedimentation rate), ESR, Highest lactic acid, Highest PCT, CRP, Cr (creatinine), Oxygenation index (PaO_2_/FiO_2_), ALB (albumin), PLT (platelet), Highest D-Dimer, Highest IL-6 (interleukin-6), IL-6, and PCT (procalcitonin). It was seen from the Lowest oxygenation index (PaO_2_/FiO_2_) that the amount of oxygen in the blood drops drastically in patients who sustain fibrosis longer (Table 1).

### CT characteristics and dynamic changes in pulmonary fibrosis

**Fig. 1:**
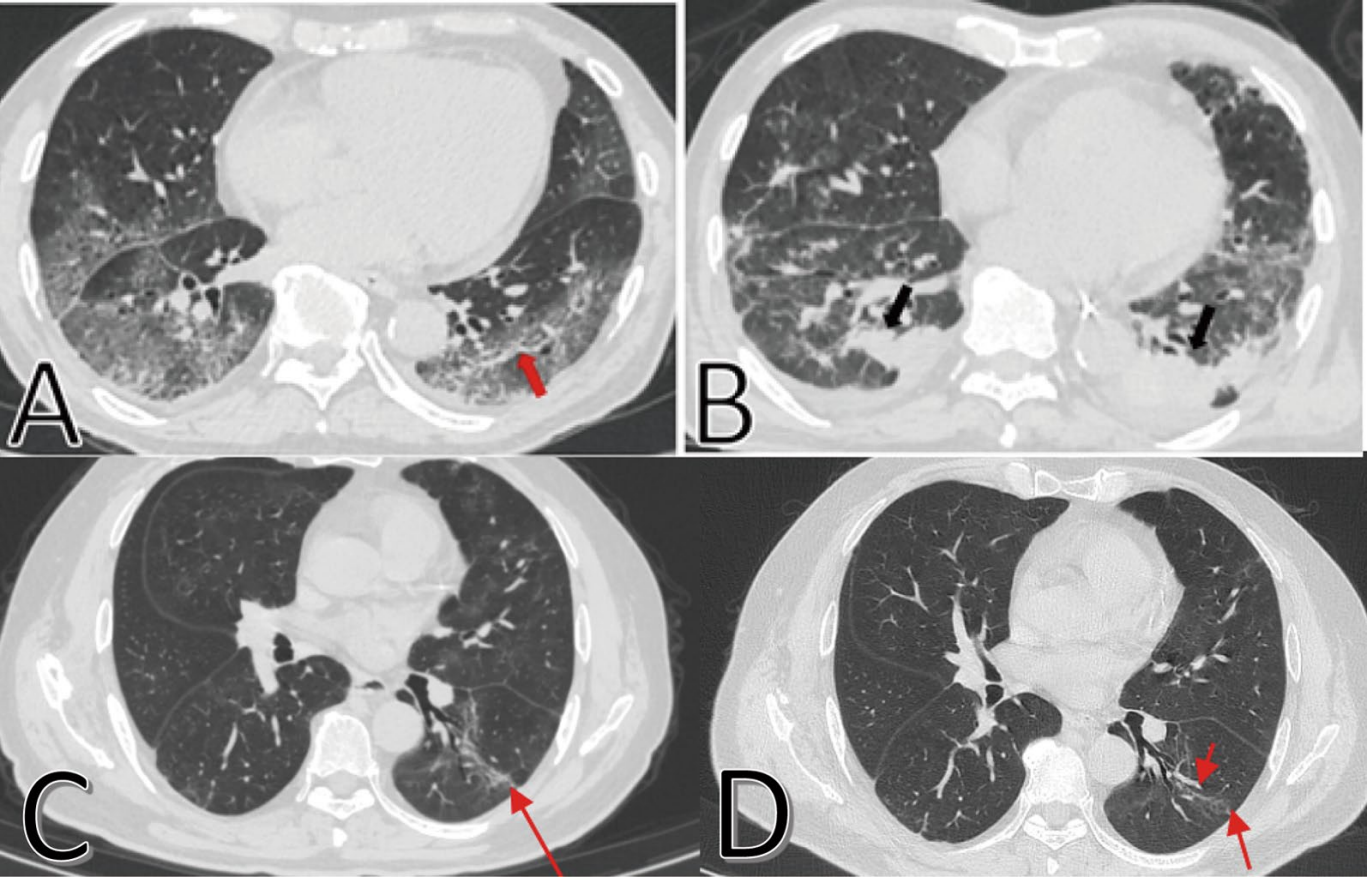
Typical CT imaging findings of a 67-year-old man with persistent pulmonary fibrosis. **A** Thin-section chest CT scan in our hospital on January 26, 2020 (9 days after symptoms onset). Chest CT imaging showed diffuse ground-glass opacities in both lungs, and a visible parenchymal band in the lower lobe of left lung (red arrow). **B** On March 23, 2020 (66 days after symptoms onset), diffuse ground-glass opacities were resolved partially in both lungs and new consolidation was observed. Irregular interfaces (black arrows) with a small amount of pleural effusion were observed in the lower lobes of both lungs. **C** On April 21, 2020 (95 days after symptoms onset), parenchymal bands and traction bronchiectasis were observed (red arrow). **D** On Jun 15, 2020 (150 days after symptoms onset), parenchymal bands and traction bronchiectasis were still observed in left lower lungs

**Fig. 2:**
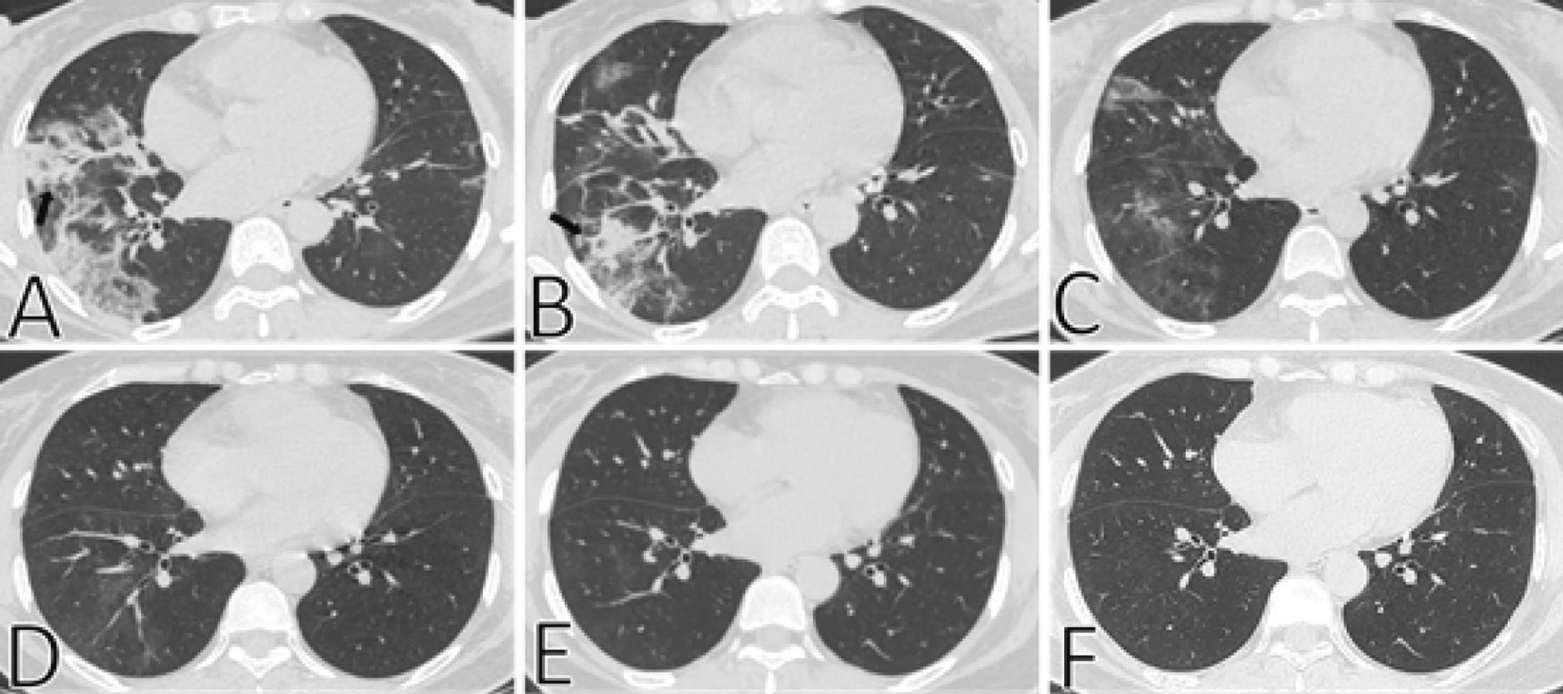
Typical CT imaging findings of a 53-year-old woman with resolved pulmonary fibrosis. **A** Thin-section chest CT scan in our hospital on February 7, 2020 (4 days after symptoms onset). Chest CT imaging showed multiple lesions in both lungs, a mass of ground-glass opacities was observed in the middle and lower lobes of the right lung, with consolidation and irregular interfaces (black arrow). **B** On February 9, 2020 (6 days after symptoms onset), the resolution of the lesion was obvious, ground-glass opacities, consolidation and irregular interfaces (black arrow) were still observed. **C** On February 12, 2020 (9 days after symptoms onset), the lung lesions were further resolved, the density of consolidation decreased. **D**, **E** On February 22, 2020 (19 days after symptoms onset) and March 14, 2020 (40 days after symptoms onset), respectively, only a little ground-glass opacities were observed in the lower lobe of the right lung, with obscure boundaries. **F** On May 22, 2020 (108 days after symptoms onset), the lesions in both lungs have been completely resolved

We analyzed the three CT scans for every patient. Ground-glass opacities (GGO), parenchymal bands, irregular interfaces, reticulation and traction bronchiectasis were the most common CT features in all COVID-19 patients. During hospitalization and follow-up, some patients had persistent pulmonary fibrosis (Fig. 1), while some patients had resolution of pulmonary fibrosis (Fig. 2). Typical CT imaging of a 67-year-old man showed diffuse GGO in both lungs, and visible parenchymal band in the lower lobe of left lung on initial CT. Diffuse GGO, consolidation and irregular interfaces with a small amount of pleural effusion were observed on the first follow-up CT. For the third (95 days after symptoms onset) and latest (150 days after symptoms onset) follow-up parenchymal bands and traction bronchiectasis were still observed in the lungs, although most lesions were resolved (Fig. 1). Typical CT imaging findings of a 53-year-old woman showed multiple lesions, a mass of GGO, consolidation and irregular interfaces on initial CT. The lesions were resolved on the 6^th^ day after onset, further resolved on the 9^th^ and 19^th^ days after onset, and completely resolved in both lungs on 108^th^ day after symptoms onset (Fig. 2).

Some patients did not follow-up along with the time extension, a total of 457 patients was included during the 0–30 days after onset in this study. During the 31–60, 61–90, 91–120 and > 120 days after onset, there were 418, 279, 207 and 79 patients included, respectively, among them, 397 (86.87%), 311 (74.40%), 222 (79.57%), 141 (68.12%) and 49 (62.03%) patients developed with pulmonary fibrosis, respectively. It is interesting to see that pulmonary fibrosis developed in COVID-19 patients could be reversed. Resolution of pulmonary fibrosis were found in 18 (4.53%), 49 (13.61%), 14 (6.31%), 30 (21.289%) and 15 (30.61%) COVID-19 patients during the 0–30, 31–60, 61–90, 91–120 and > 120 days after onset, respectively (Table 2). There was a total of 397 patients had lung fibrosis, among them, 126 patients had pulmonary fibrosis reversed, the median time of resolution of pulmonary fibrosis was 70.79 ± 37.67 days (median: 59 days, range: 8–148 days). In contrast, there were 271 patients who still had pulmonary fibrosis at the last follow-up. The overall lung fibrosis and overall resolution of fibrosis percentage were 86.87% and 31.74%, respectively (Table 2). The CT imaging features including ground-glass opacities, meshwork, parenchymal bands, irregular interface and traction bronchiectasis of the COVID-19 patients at different stages were analyzed meanwhile. Ground-glass opacities and parenchymal bands are most common CT features in these patients (Table 3).

**Table 2:** Dynamic changes of pulmonary fibrosis in 457 patients at different stages after the onset of COVID-19

COVID-19 Patients	Days after onset				
	0–30	31–60	61–90	91–120	> 120
Patients included	457	418	279	207	79
Patients with pneumonia	428 (93.65%)	397 (94.98%)	272 (97.49%)	202 (97.58%)	77 (97.47%)
Patients with pulmonary fibrosis	397 (86.87%)	311 (74.40%)	222 (79.57%)	141 (68.12%)	49 (62.03%)
Patients with resolution of pulmonary fibrosis	18 (4.53%)	49 (13.61%)	14 (6.31%)	30 (21.28%)	15 (30.61%)
Overall pulmonary fibrosis	397 (86.87%)				
Overall resolution of fibrosis	126 (31.74%)

**Table 3:** CT imaging features in 457 patients at different stages after the onset of COVID-19

COVID-19 Patients	Days after onset				
	0–30	31–60	61–90	91–120	> 120
Ground-glass opacities (GGO)	88.66% (352/397)	90.76% (275/303)	91.41% (149/163)	75.58% (65/86)	65.31% (32/49)
Meshwork	30.73% (122/397)	13.53% (41/303)	14.11% (23/163)	6.98% (6/86)	10.20% (5/49)
Parenchymal bands	82.37% (327/397)	94.72% (287/303)	95.71% (156/163)	97.67% (84/86)	93.88% (46/49)
Irregular interface	26.45% (105/397)	25.41% (77/303)	25.15% (41/163)	22.09% (19/86)	28.57% (14/49)
Traction bronchiectasis	5.54% (22/397)	14.52% (44/303)	19.02% (31/163)	16.28% (14/86)	14.29% (7/49)

### Establishment of prediction model of the persistence of pulmonary fibrosis

A total of 56 features (Table 1) were collected from each patient. After performing the ANOVA, a set of 35 statistically significant (*p* < 0.05) remained for developing the prediction model. However, features related to treatment measures and temporal patterns were not considered for model development since those features are determined by the physicians based on the condition of the patients and thereby are dependent variables.

**Fig. 3:**
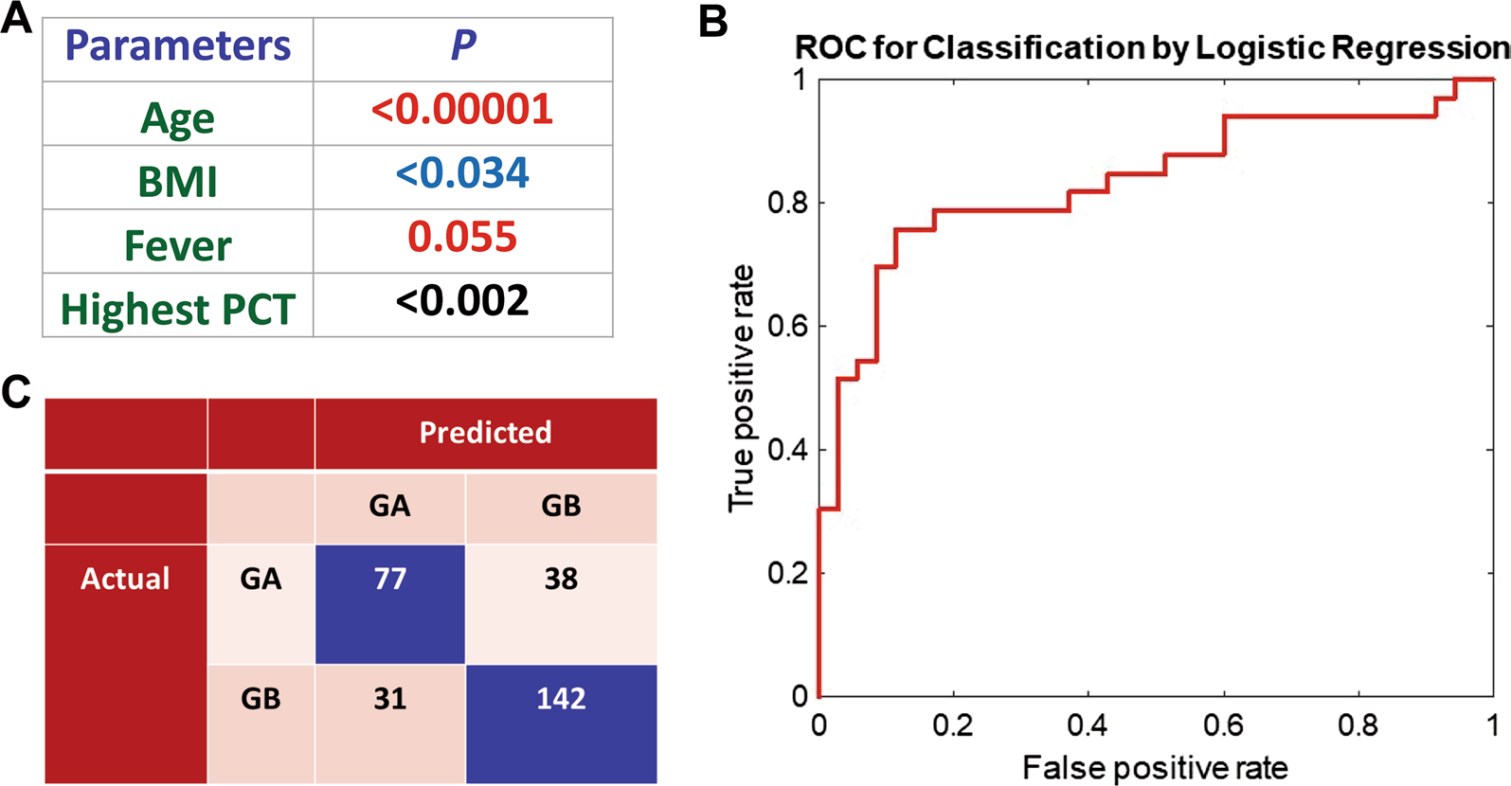
Prediction model of the persistence of pulmonary fibrosis. **A** Identified 4 Parameters that can distinguish between two groups. **B** The confusion matrix of the five-fold cross validation was used to validate the performance of the model. **C** Receiver operating characteristic curve which was used to evaluate the accuracy, Positive Predictive Value (PPV), Negative Predictive Value (NPV) and Sensitivity and Specificity of the model. True positive rate = Sensitivity; True negative rate = Specificity

Among the statistically significant independent variables, it was observed that Age, BMI, Fever, and Highest PCT were the predictive factors of sustained fibrosis even after 90 days from onset (Fig. 3A). Among the 289 patients, 288 patients had complete data for these four parameters that were used to develop a predictive model based on the Logistic regression method. The performance of the model was validated by fivefold cross validation and evaluated by the ROC, accuracy, PPV, NPV, Sensitivity and Specificity. Cross validation ensures the consistency of the model’s performance while reducing model bias and variance. The confusion matrix of the five-fold cross validation is presented in Fig. 3B. An average AUC (Area under the ROC Curve) of 0.84 was obtained from fivefold cross validation that affirms good reliability of the predictive model. The accuracy, PPV, NPV, Sensitivity and Specificity of the model were 76%, 71%, 79%, 67%, and 82%, respectively (Fig. 3C).

### Pulmonary function of patients with pulmonary fibrosis and with no fibrosis/resolution of pulmonary fibrosis after 90 days from onset

To observe the effect of pulmonary fibrosis on lung function, 33 patients from Group A (GA) and 114 patients from Group B (GB) underwent pulmonary function testing after 90 days from onset. Six patients (18.18%) in GA and 26 patients (22.81%) in GB had a pulmonary function abnormality. Two patients (6.06%) in GA and 5 patients (4.39%) in GB were diagnosed with the obstructive pulmonary disorder. The restrictive disorder was found in 1 patient (3.03%) in GA and 8 patients (7.02%) in GB. Besides, 3 patients (9.09%) in GA and 13 patients (11.40%) in GB had small airway disorders. MEF 25 decrease was found in 12 patients (36.36%) in GA and 46 patients (40.35%) patients in GB. 2 patients (6.06%) in GA and 8 patients (7.02%) in GB had a pulmonary diffusion abnormality (Table 4). Overall, 18 patients (54.55%) and 76 patients (66.67%) revealed abnormal condition in pulmonary function test. It is interesting to see that the ratio of abnormal lung function, obstructive disorder, restrictive disorder, small airway disorder, MEF 25 decrease, abnormal lung diffusion and overall abnormalities in lung function test did not differ on a statistically significant level (p > 0.05) between the two groups.

**Table 4:** Pulmonary function test results and clinical symptoms for two groups of COVID-19 patients with pulmonary fibrosis

	Group A^a^ (N = 33)	Group B^b^ (N = 114)	P value
Overall abnormalities ratio in pulmonary function test	54.55% (18/33)	66.67% (76/114)	0.2841
Abnormal lung function ratio^c^	18.18% (6/33)	22.81% (26/114)	0.7433
Obstructive disorder ratio	6.06% (2/33)	4.39% (5/114)	0.9471
Restrictive disorder ratio	3.03% (1/33)	7.02% (8/114)	0.6679
Small airway disorder ratio	9.09% (3/33)	11.40% (13/114)	0.9535
MEF 25 decrease^d^	36.36% (12/33)	40.35% (46/114)	0.8333
Abnormal lung diffusion function	6.06% (2/33)	7.02% (8/114)	0.8413
The patients with persistent PFT abnormalities and ongoing symptoms	44.44% (8/18)	44.74% (34/76)	0.8094
Severe/critical patients ratio	3.03% (1/33)	24.56% (28/114)	0.0128
Symptomatic patients ratio	30.30% (10/33)	43.86% (50/114)	0.2324
Dyspnea	3.03% (1/33)	1.75% (2/114)	0.8084
exercise limitation	6.06% (2/33)	8.77% (10/114)	0.8887
Cough	18.18% (6/33)	14.91% (17/114)	0.8546
Fatigue	9.09% (3/33)	21.05% (24/114)	0.1910
chest tightness	3.03% (1/33)	7.89% (9/114)	0.5587
Hyposmia	0% (0/33)	1.75% (2/114)	0.9306
Ongoing O_2_ requirement	0	0	–

Lung function tests were conducted in about 3 months after the onset

^a^Patients who either had no lung fibrosis, or their lung fibrosis disappeared within 90 days after onset

^b^Patients who still had lung fibrosis after 90 days from onset

^c^The patients were evaluated the lung function using spirometry to obtain relevant indices including first second exhalation volume (FEV1), forced vital capacity (FVC), FEV1/FVC%, forced expiratory flow rate at 50% and 75% of FVC (FEF50%, FEF75%), forced expired flow at 25–75% of FVC (FEF25-75%), inspiratory reserve volume (IRV), tidal volume (TV) and expiratory reserve volume (ERV)

^d^MEF 25, maximal expiratory flow after 25% of the forced vital capacity has not been exhaled

GB had more patients with critical and severe symptoms than GA (24.56% vs 3.03%) which is statistically significant at p < 0.02. As discussed earlier, patients presented with critical and severe symptoms tend to be more vulnerable to sustaining lung fibrosis even after 90 days (Table 4). There is no any clinical correlation between persistent fibrosis and PFT abnormalities on a statistically significant level was observed. As presented in Table 4, ten patients (30.30%) in GA and 50 patients (43.86%) in GB had an ongoing clinical syndrome. Only one patient (3.03%) in GA and two patients (1.75%) in GB had dyspnea. Two patients (6.06%) in GA and ten patients (8.77%) in GB experienced exercise limitation. Six patients (18.18%) in GA and 17 patients (14.91%) in GB had a cough. Only one patient (3.03%) in GA and nine patients (7.89%) in GB experienced chest tightness. Only two patients (1.75%) in GB experienced hyposmia, whereas no patient in GA reported this symptom. No patients in either group had ongoing O_2_ requirements. Interestingly, a higher number of people in GB (21.05%) in comparison to 9.09% people in GA reported fatigue, although the observation is not statistically significant. In addition, 18 patients in GA and 76 patients in GB had persistent PFT abnormalities, among which eight (44.44%) patients in GA and 34 (44.74%) patients in GB had ongoing symptoms.

## Discussion

Data from previous coronavirus infections such as SARS, as well as emerging data from the COVID-19 pandemic, suggest there could be substantial fibrotic consequences following SARS-CoV-2 infection [5, 19–22]. Given the huge numbers of individuals infected by SARS-CoV-2, it is important to identify and predict the occurrence of pulmonary fibrosis in the survivor population after discharge.

In our study, thin-section CT scans obtained in hospitalized or discharged patients have shown that fibrosis occurred in a large proportion of the COVID-19 patients, and it occurred in more than a half even for the subset of patients after 120 days from onset. Numerous similarities were found among SARS-CoV coronavirus, MERS coronavirus and SARS-CoV-2. In an early follow-up study of patients with SARS, 62% patients revealed CT evidence of pulmonary fibrosis at a mean follow-up duration of 37 days after hospital discharge [5], whereas in a follow-up study of patients with MERS, 33% had radiographic evidence of pulmonary fibrosis [4]. In view of this, the infection of SARS-CoV-2 has a high incidence of pulmonary fibrosis that is comparable to those of SARS and MERS.

Clinically, patients with fibrosis after a long time from the onset were significantly older, with higher BMI and significantly higher proportions of fever and severe/critical COVID cases than those without fibrosis. These results implied that fibrosis was likely to be more common in elderly, obese and severe/critical patients, similar to those patients with SARS [5], and COVID-19 patients with fever are more vulnerable to developing pulmonary fibrosis. Besides, patients with pulmonary fibrosis needed a much longer time to turn the RNA of SARS-CoV-2 negative in their bodies implying the fact that the longer the virus lived in vivo, the more damage it did to the patient's lungs. Meanwhile, it is interesting to observe that a delay of on average ~ 2 days in hospital admission following the onset of the symptoms had a significant detrimental effect on the patients with pneumonia, suggesting the importance of seeking medical advice for the COVID-19 patients in time.

There were statistically significant differences in some of the laboratory findings between the fibrotic and non-fibrotic groups, include some extreme lab values, lowest PaO2/FiO2, Lowest CD4 cell, Highest CRP, Highest ESR, Highest lactic acid, Highest PCT, Highest D-Dimer, Highest IL-6. These differences of laboratory findings may help clinicians more readily look for and make the diagnosis of pulmonary fibrosis in COVID-19 patients.

The pulmonary fibrosis of COVID-19 patients in our study was identified by reviewing the CT scans. In many previous studies, this method was also used to diagnose the lung damage resulted from viral pneumonia, such as lung fibrosis [4, 9, 14, 22, 23]. Thin-section CT scans from all the patients showed that evidence of fibrosis was found in almost half of patients that was consistent with the findings of some previous studies in COVID-19 patients [9, 14, 24]. We found that pulmonary fibrosis developed in COVID-19 patients could be reversed in a part of the population. The pulmonary fibrosis in some patients was persistent during the follow-up period. Therefore, it is necessary to follow-up these patients for a longer time to assess the consequence of persistent pulmonary on them.

At one-year follow-up of the survivors from SARS, pulmonary function testing showed a pulmonary function abnormality in a subset of the patients, who suffered from poor quality of life after discharge [25]. In this study, the infection of SARS-CoV-2 has a high incidence of pulmonary fibrosis. However, it is interesting to see that about half of COVID-19 patients suffered from abnormal lung function after 90 days from onset, and the ratio of abnormal lung function did not differ on a statistically significant level between the fibrotic and non-fibrotic groups. These results suggested that the pulmonary function of less than half of COVID-19 patients with or without pulmonary fibrosis could turn to normal condition after three months from onset.

A previous study indicated that early treatment with steroids was well tolerated and associated with rapid and significant improvement for patients with persistent inflammatory interstitial lung disease following SARS-CoV-2 infection [26]. In our study, more than 30% of hospitalized COVID-19 patients in group B received steroid treatment in the early stage of treatment, which might play into the resolution of some of the CT scan findings, aid in the initial treatment of this patient population, and prevent long-term irreversible lung damage.

Significantly, we established a prediction model for the prediction of the persistence of pulmonary fibrosis in this study for early diagnosis. It was observed that Age, BMI, Fever, and Highest PCT were predictive factors of sustained fibrosis even after 90 days. The patients with a high risk to develop persistent pulmonary fibrosis deserve special attention, anti-fibrosis drugs can be considered in the early stage of treatment to prevent further damage to the lungs of such patients.

Nevertheless, this study has some limitations. First, the follow-up time for these patients is not long enough, and it is unknown whether the pulmonary fibrosis will permanently remain. Second, there is no histologic confirmation of fibrosis in any of the patients, although the signs on thin-section CT scans are convincing. Third, COVID-19 patients admitted to our hospital in this study were mostly imported from abroad or from outside the province. Most patients were non-residents of Shenzhen and most patients left Shenzhen after their condition improved. Therefore, the loss rate of follow-up in this study is high, a certain part of discharged patients was lost, leaving only 79 patients that are included during > 120 days after onset. Fourth, this study is centered mainly on the development of lung fibrosis lesions and on their resolution. However, it did not take into due consideration important clinical parameters to confirm that CT images. Fifth, the follow-up did not take in due consideration the levels of urinary hydroxyproline (which derive from the collagen catabolism) obtained from urine analysis, which may demonstrate the true existence of an increased lung collagen resorption during the fibrosis resolution. Sixth, this study lacks symptom assessment at the follow-up to evaluate whether persistent imaging abnormalities correlate with ongoing symptom burden.

## Conclusions

Persistent pulmonary fibrosis was more likely to develop in patients with older age, higher BMI, severe/critical condition, fever, long time to turn the viral RNA negative in vivo, pre-existing disease and delay in hospital admission. Pulmonary fibrosis occurred in a large proportion of the COVID-19 patients, even for the patients after 120 days from onset. However, fibrosis developed in COVID-19 patients could be reversed in about a third of the patients after 120 days from onset. The pulmonary function of less than half of COVID-19 patients with pulmonary fibrosis could turn to normal condition after three months from onset. An effective prediction model with an average AUC of 0.84 was established to predict the persistence of pulmonary fibrosis in COVID-19 patients for early diagnosis. By doing this, we hope to deliver appropriate clinical care and in time design interventional trials to the patients with a high risk to develop persistent pulmonary fibrosis. Future follow-up studies with a longer follow-up period would be necessary to confirm our findings and better determine the long-term outcomes of patients who recovered from COVID-19.

## Data Availability

The datasets used and/or analyzed during the current study are available from the corresponding author on reasonable request.

## Abbreviations

COVID-19: The Coronavirus Disease 2019
SARS: Severe acute respiratory syndrome
MERS: Coronavirus and Middle East respiratory syndrome
CT: Chest computed tomography
qRT-PCR: Quantitative reverse-transcription polymerase chain reaction
ROC: Receiver operating characteristic
PPV: Positive predictive value
NPV: Negative predictive value
AUC: Area under the ROC curve
